# Photocurable Glycerol- and Vanillin-Based Resins for the Synthesis of Vitrimers

**DOI:** 10.1021/acsapm.2c00914

**Published:** 2022-08-02

**Authors:** Sigita Grauzeliene, Marius Kastanauskas, Vaidas Talacka, Jolita Ostrauskaite

**Affiliations:** † Department of Polymer Chemistry and Technology, Kaunas University of Technology, Radvilenu Rd. 19, Kaunas LT-50254, Lithuania; ‡ AmeraLabs, Kestucio str. 6A, Kaunas LT-44320, Lithuania

**Keywords:** vitrimer, glycerol, vanillin, shape-memory, self-healing, recycling, DLP 3D printing

## Abstract

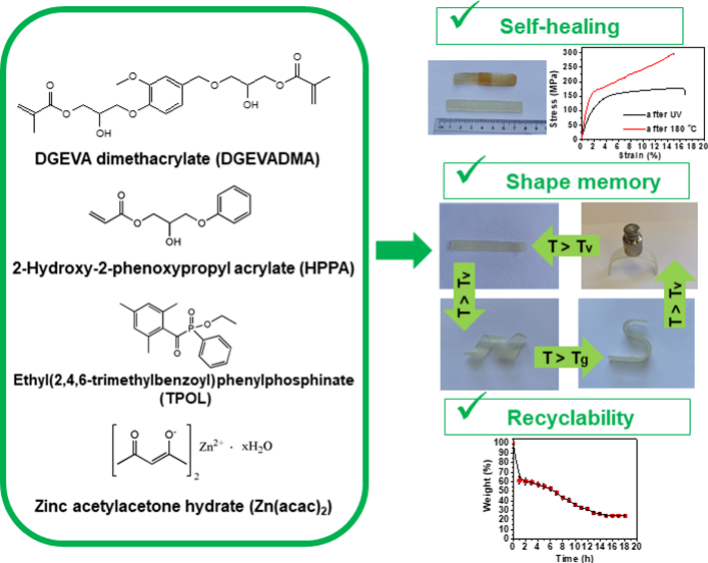

In this study, photocurable resins based on glycerol and vanillin were designed, synthesized, and applied to digital light processing three-dimensional (3D) printing and vitrimeric abilities such as shape-memory, self-healing, and recyclability have been investigated. First, photocurable resins were prepared and synthesized by combining renewable resources and photocuring as an environmentally friendly strategy for the synthesis of vitrimers. Afterward, the most suitable resin for optical 3D printing was selected by photorheometry, and the thermal and mechanical properties of the resulting polymers were tested. Furthermore, by activating dynamic transesterification reactions at elevated temperatures, the photocured polymer exhibited self-healing, recyclability, and shape-memory properties. The vitrimer with a weight ratio of 8:2 of glycerol- and vanillin-based monomers demonstrated a welding efficiency of tensile strength up to 114.12%, 75% recyclability by alcoholysis, and shape-memory properties above and below two glass transition temperatures.

## 1 Introduction

The global production of thermosets is expanding in recent years with the rapid usage of fossil feedstocks, which contribute to the amount of plastic waste in the environment since they cannot be recycled or reprocessed after use.^1^ To solve this problem, scientists have introduced dynamic cross-links that can rearrange their network by exchange reactions and can have shape-memory, can have self-healing properties, and can be reprocessable.^2^ The first dynamic cross-links were presented by French researcher Ludwik Leibler, and they were named “vitrimers” since they flow like vitreous silica following the Arrhenius law.^3^ They behave as cross-linked thermosets at operating temperatures, while at elevated temperatures, the exchange reactions speed up, making flow possible, while the number of chemical bonds and cross-links remains the same.^4,5^ Due to this behavior, vitrimers can be repaired, reshaped, and reprocessed by injection, extrusion, or three-dimensional (3D) printing.^6^ Plenty of structural and fast-acting devices such as actuators for soft robotics, which even could be repaired or reprocessed, can be manufactured leading to sustainable development and environmental protection.^7,8^

Frequently used optical 3D printing technologies, such as stereolithography (SLA) and digital light processing (DLP), have advantages such as high resolution and accuracy, good surface finish, and high fabrication speed.^9^ However, the resin must consist of a monomer, a photoinitiator, and a diluent that adjusts the viscosity^10^ as the resin must have a relatively low or medium viscosity that meets the requirements of a 3D printer.^11^ Acrylate- and methacrylate-based resins are the most commonly used for 3D printing due to the high polymerization rate and commercially available monomers.^11^ Acrylate-based resins having hydroxyl and ester groups have been used to prepare vitrimers by optical 3D printing, which after the 3D printing process can undergo thermoactivated transesterification reactions.^6,8,12−17^ These vitrimers were reshapable, repairable, and recyclable, which contributed to alleviating the environmental challenges associated with the continuous increase in the consumption of 3D printing materials. The most commonly used monofunctional acrylates in the 3D printing of vitrimers are 2-hydroxy-2-phenoxypropyl acrylate (HPPA), as has hydroxyl and ester groups that are essential in the transesterification reactions, and benzene ring, which can increase thermal and mechanical properties. However, the most commonly used cross-linker is diacrylate with the fragment of bisphenol A, which is a pollutant of water and provocative of various metabolic and endocrine diseases.^18−20^

**Figure 1:**
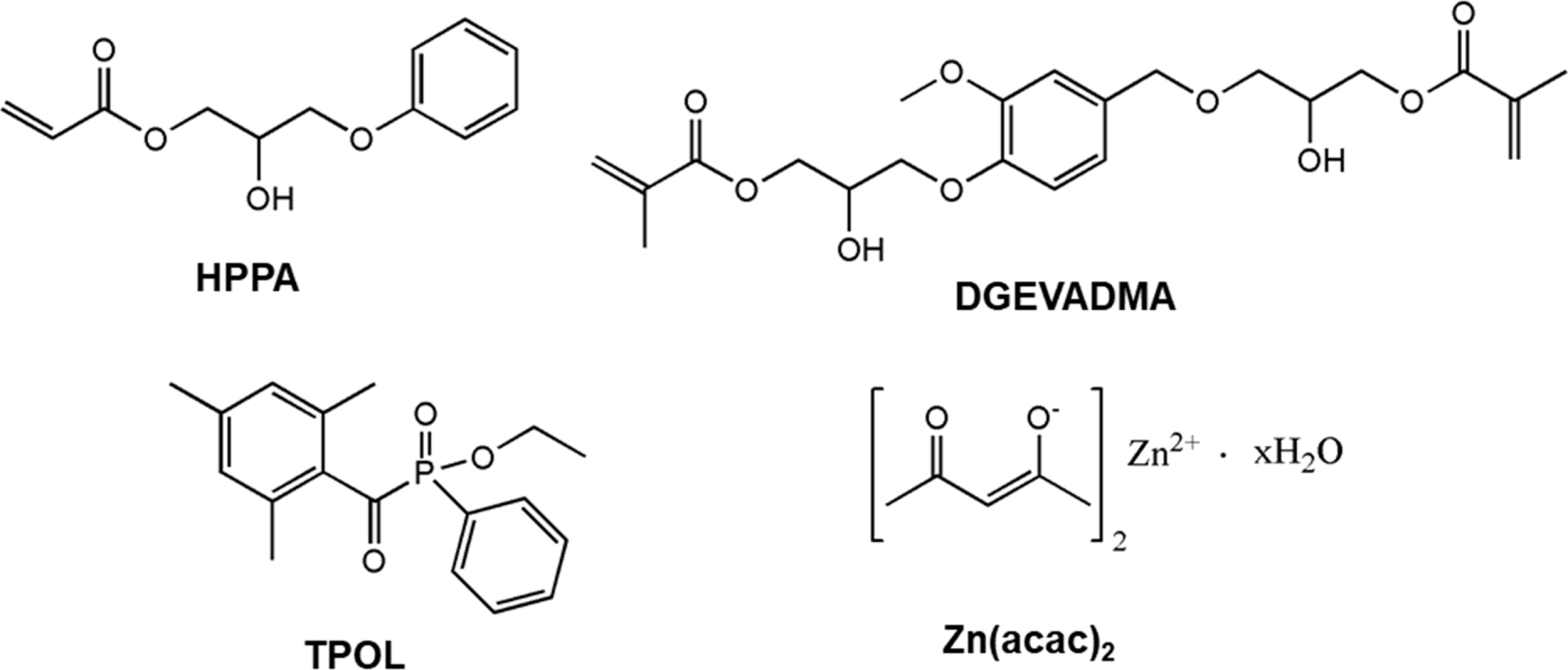
Chemical structures of 2-hydroxy-2-phenoxypropyl acrylate (HPPA), DGEVA dimethacrylate (DGEVADMA), ethyl(2,4,6-trimethylbenzoyl)phenylphosphinate (TPOL), and zinc acetylacetone hydrate (Zn(acac)_2_).

In this study, UV-curable glycerol- and vanillin-based resins are presented for the synthesis of vitrimers. Vitrimers were synthesized using an environmentally friendly strategy by combining monomers in various ratios with a photoinitiator and a transesterification catalyst and irradiating them under solvent-free conditions. 2-Hydroxy-2-phenoxypropyl acrylate (HPPA) (Figure 1) was chosen as the most commonly used monofunctional monomer for vitrimer synthesis by transesterification reactions. Furthermore, HPPA has a fragment of glycerol, which is the main component of triglycerides, found in animal fat, vegetable oil, or crude oil.^21^ Glycerol can be derived from biodiesel production from vegetable oils and animal fats^22^ and has even been used for the production of biodegradable plastics already.^23−25^ 2-Hydroxy-3-[[4-[2-hydroxy-3-[(2-methyl-1-oxo-2-propen-1-yl)oxy]propoxy]-3-methoxyphenyl]methoxy]propyl 2-methyl-2-propenoate (DGEVA dimethacrylate, DGEVADMA) was chosen as a cross-linker due to hydroxyl and ester functional groups and is reported here for the first time in UV-curable resins for vitrimer synthesis. DGEVA dimethacrylate can be a good alternative to the most widely used cross-linker with the bisphenol A fragment as it has a vanillin-based backbone with high rigidity and thermal stability.^26^ Vanillin-based polymers have also shown antibacterial and antifungal properties,^27^ which is relevant nowadays. DGEVA dimethacrylate was reported in UV-curable resins with thiols as having shape-memory and antimicrobial properties.^28^ DGEVA dimethacrylate is attractive for vitrimer synthesis due to the mentioned properties and that can be produced from the second most abundant natural phenolic polymer lignin.^29^ Therefore, this study focuses on the development of new UV-curable resins based on glycerol and vanillin for vitrimer synthesis, which would be suitable for optical 3D printing on demand. The photoinitiator ethyl(2,4,6-trimethylbenzoyl)phenylphosphinate (TPOL) was used due to the photobleaching effect allowing to get transparent coatings.^30^ One of the most commonly used catalysts, zinc acetylacetone hydrate (Zn(acac)_2_),^31^ was chosen as the transesterification catalyst as it was soluble in the selected acrylate monomers. For the first time, cross-linking kinetics and rheological properties of resins based on glycerol and vanillin were monitored. Stress relaxation experiments revealed that, after UV curing, the dynamic networks could quickly undergo a thermoactivated network topology rearrangement, showing suitability of DGEVA dimethacrylate as the cross-linker of HPPA. The desired properties of self-healing, shape-memory, and recyclability have been achieved due to the incorporation of DGEVA dimethacrylate in the resins with HPPA. The resin based on glycerol and vanillin was used to form vitrimeric 3D structures by DLP 3D printing.

## 2 Materials and Methods

### 2.1 Materials

2-Hydroxy-2-phenoxypropyl acrylate (HPPA), zinc acetylacetone hydrate (Zn(acac)_2_), and 2,5-bis(5-*tert*-butylbenzoxazol-2-yl)thiophene were purchased from Merck. 2-Hydroxy-3-[[4-[2-hydroxy-3-[(2-methyl-1-oxo-2-propen-1-yl)oxy]propoxy]-3-methoxyphenyl]methoxy]propyl 2-methyl-2-propenoate (DGEVA dimethacrylate, DGEVADMA) was purchased from Specific Polymers. Ethyl(2,4,6-trimethylbenzoyl)phenylphosphinate (TPOL) was purchased from Fluorochem. All chemicals were used as received.

### 2.2 Preparation of UV-Curable Resins

The resins were prepared by dissolving 5 mol % of transesterification catalyst Zn(acac)_2_ in HPPA at 70 °C. After cooling the resin to room temperature, different amounts of DGEVADMA and 3 mol % of TPOL as the photoinitiator were added and stirred at room temperature. The resin codes consist of a number expressing the amount and the first letter of monomer abbreviation, e.g., 20D/80H is a resin with the weight ratio of 8:2 of monomer HPPA and cross-linker DGEVADMA.

The viscosity of glycerol- and vanillin-based resins was measured with a rheometer MCR302 from Anton Paar (Graz, Austria) with a plate/plate accessory (15 mm diameter of the top plate) using a shear rate from 0.001 to 50 s^–1^ at 25 °C temperature.

The resins were UV-cured in a Teflon mold of (70 × 10 × 1) ± 0.01 mm under a 500 W Helios Italquartz GR.E UV lamp at a wavelength of 250–450 nm and intensity of 310 mW·cm^–2^ for 2 min until the solid polymer was obtained.

### 2.3 DLP 3D Printing

DLP 3D printing was performed on a Phrozen Sonic Mini 4K 3D printer (desktop LCD/LED, Hsinchu, Taiwan) with a 405 nm light source. A layer of 50 μm was exposed for 12 s. 2,5-Bis(5-*tert*-butylbenzoxazol-2-yl)thiophene (0.08%) as the UV blocker was used in the resin. The “City” structure was printed to test the accuracy and capability for 3D printing of the resin. Rectangular samples with dimensions of (60 × 20 × 2) ± 0.01 mm were printed to test shape-memory properties. All samples were cleaned with isopropyl alcohol for 20 min and post-cured in a UV chamber (LED light source: λ = 365 nm (45 W), 380 nm (25 W), and 395 nm (70 W)) for 20 min.

### 2.4 Characterization Techniques

Photocuring kinetics of the resins was studied by using an MCR302 (Graz, Austria) rheometer from Anton Paar with a plate/plate accessory and OmniCure S2000 curing system from Lumen Dynamics Group Inc. (Mississauga, Ontario, Canada). The diameters of the bottom glass plate and the top steel plate were 38 and 15 mm, respectively. The tests were carried out at 25 °C and a thickness of the resins of 0.1 mm. The resins were irradiated with UV–vis radiation with a wavelength of 250–450 nm and an intensity of 9.3 W·cm^–2^ through a bottom glass plate. A shear test was performed using a frequency of 10 Hz and a strain of 0.5%. The values of storage modulus *G*′, loss modulus *G*″, and complex viscosity η* of the resins were determined after 120 s of irradiation. The gel point *t*_gel_ was identified as the crossover point of the *G*′ and *G″* modulus curves. The shrinkage was defined as the difference between the gap before and after cross-linking.

Fourier transformation infrared spectroscopy (FT-IR) reflection spectra were recorded using a Spectrum BX II FT-IR spectrometer (PerkinElmer, Llantrisant, UK) in the wavenumber range of 650–4000 cm^–1^.

The yield of the insoluble fraction was determined by extracting polymer samples (0.2 g) with acetone for 24 h in a Soxhlet extractor. The samples were dried until the constant weight and the yield of the insoluble fraction were calculated as the difference between the weight of the polymer sample before and after extraction and drying.

The elongation at break, tensile strength, and Young’s modulus were estimated by a tensile test on a Testometric M500-50CT machine (Testometric Co., Ltd., Rochdale, UK) with HDFF100 grips according to the standard ISO 527-3. The samples with dimensions of (70 × 10 × 1) ± 0.01 mm were tested with a crosshead speed of 5 mm/min. Five measurements were made to obtain the average values. The variation in the experimental results did not exceed 5% within the group.

Topology freezing temperature (*T*_v_) was determined by the stress relaxation experiments on an MCR302 rheometer from Anton Paar (Graz, Austria). The samples were equilibrated to the selected measurement temperature (160, 180, and 200 °C), a specified constant normal force of 20 N for 20 min and 5% step strain was applied, and the decreasing stress was recorded over time.

The glass transition temperature (*T*_g_) was determined by dynamic mechanical thermal analysis (DMTA) with a MCR302 rheometer from Anton Paar (Graz, Austria). The samples with dimensions of (10 × 10 × 1) ± 0.01 mm were tested in a shear mode from −20 to 130 °C at a rate of 2 °C/min. *T*_g_ was defined as the maximum of the tan δ curve peak.

The thermal stability of the polymers was determined with a PerkinElmer TGA 4000 apparatus (Llantrisant, UK) with a heating rate of 20 °C·min^–1^ and a nitrogen flow rate of 100 mL/min.

### 2.5 Self-Healing Experiment

Photocured samples with the dimensions of (10 × 10 × 1) ± 0.01 mm were cut into two equal pieces, then brought in contact with each other, and heated at 180 °C for 1 h. Rejoined samples were mechanically tested with a Testometric M500-50CT machine (Testometric Co., Ltd., Rochdale, UK).

### 2.6 Shape-Memory Experiments

Shape-memory experiments were carried out with the DLP 3D printed sample with dimensions of (60 × 20 × 2) ± 0.01 mm. The first shape was obtained by heating the sample at 120 °C (above *T*_v_), transforming to the desired shape, and cooling to 40 °C (above the *T*_g_). The second shape was obtained by heating the sample at 80 °C (above the *T*_g_) due to transforming and cooling the sample below room temperature with an ice bath. By heating again above the *T*_v_ and cooling the sample below room temperature, the third shape was obtained. The permanent shape was regained again by heating above the *T*_v_.

### 2.7 Recyclability Experiments

The chemical degradation was tested by immersing the samples (0.1 g) into vials with ethanol (10 mL) at 180 °C. The samples were taken out every hour, dried, and weighed. The relative weight was calculated as the difference between the mass before and after degradation. At least three samples were studied to get an average value.

## 3 Results and Discussion

### 3.1 Kinetics of Photocuring

**Figure 2:**
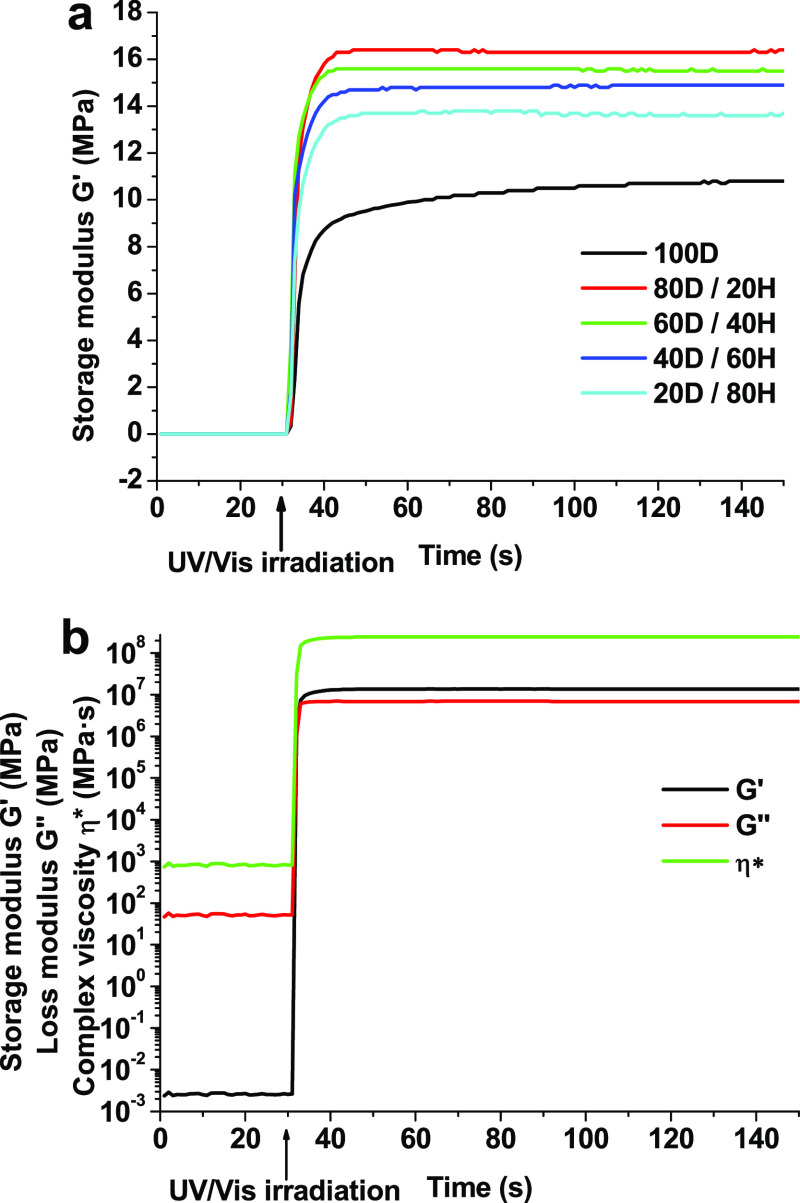
Curves of the storage modulus *G*′ versus irradiation time of resins containing different amounts of HPPA (a), curves of the storage modulus *G*′, loss modulus *G″*, and complex viscosity η* of UV-cured resin 20D/80H (b).

Photorheometry is an essential tool for monitoring rheological parameters, such as curing rate, viscosity, and shrinkage, which are important for the optical 3D printing process.^32^ This analysis is relevant when the resin transits from liquid to solid during the photocross-linking process. Viscosity shows how the resin is capable of renewing the feedstock material when the platform moves to form a layer during the optical 3D printing process, and the viscosity values of conventional resins are mainly in the range of 200–1500 mPa·s.^33^ Meanwhile, glycerol- and vanillin-based resins had viscosity in the range of 405–16,278 mPa·s (Table 1) and the increase in the monomer HPPA with the fragment of glycerol reduced the viscosity. After keeping the resins for 3 months in the dark, no significant changes in the viscosity were observed. Table 2 represents the data collected from the measurements of rheological parameters such as the storage modulus (*G*′), loss modulus (*G*″), complex viscosity (η*), and the shrinkage. The resin with the neat vanillin derivative had a *G*′ modulus value of 11.42 MPa, which was the lowest among all resins tested and was comparable to those of DGEVADMA polymers with dithiol tested by the same real-time photorheometry method (5.47–14.92 MPa).^28^ This observation shows that the incorporation of the monomer HPPA with the fragment of glycerol increased the rigidity of the final polymer network as a result of the denser cross-linked network. The curves of the storage modulus *G*′ versus the irradiation time of resins containing different amounts of HPPA are presented in Figure 2a. No correlation with the *G*′ modulus was noticed when the amount of HPPA was increased in the resin, although the *G*″ modulus representing the energy dissipated as heat^32^ also had one of the lowest values among the resins (6.23 MPa). The η* values increased in all resins by incorporation of HPPA and showed the resistance to flow as a function of the angular frequency. The shrinkage of the resins containing the glycerol fragment was higher (11.5–14.5%) than the neat DGEVADMA resin (10.5%) because the lower viscosity and the *t*_gel_ values had no influence since the gelation of all resins started after 2 s of irradiation. According to the data from the literature, the greater part of the resin prepared for vitrimer synthesis by optical 3D printing generally consists of the monomer and the smaller part consists of the cross-linker.^15^ Therefore, the resin 20D/80H with the lowest viscosity of 405 mPa·s and the highest amount of glycerol monomer HPPA was selected for further investigation of vitrimeric properties. Figure 2b shows that the gelation of resin 20D/80H started at 2 s after resin irradiation, indicating a growth of the chain size and network formation, and the values of *G*′, *G*″, and η* increased immediately, reaching values of 13.55 MPa, 6.16 MPa, and 237.07 MPa·s, respectively.

**Table 1:** Composition of Glycerol- and Vanillin-Based Resins

resin	amount of DGEVADMA [wt %]	amount of HPPA [wt %]	amount of TPOL [mol %]	amount of Zn(acac)_2_ [mol %]	viscosity [mPa·s]
100D	100		3	5	16,278 ± 592
80D/20H	80	20			6101 ± 109
60D/40H	60	40			2609 ± 211
40D/60H	40	60			1315 ± 260
20D/80H	20	80			405 ± 6

**Table 2:** Real-Time Photorheometry Data of Glycerol- and Vanillin-Based Resins

resin	storage modulus *G*′ (MPa)	loss modulus *G″* (MPa)	complex viscosity η* (MPa·s)	shrinkage (%)
100D	11.42 ± 0.58	6.23 ± 0.40	212 ± 17	10.5 ± 0.5
80D/20H	16.11 ± 2.95	8.26 ± 0.07	288 ± 3	11.5 ± 0.5
60D/40H	14.87 ± 0.68	7.16 ± 0.82	263 ± 4	12.0 ± 0.0
40D/60H	13.59 ± 1.30	7.39 ± 0.89	247 ± 11	12.5 ± 0.5
20D/80H	13.55 ± 0.11	6.16 ± 0.80	237 ± 7	14.5 ± 0.5

### 3.2 Characterization of the Cross-Linked Polymer Structure

The chemical structure of glycerol- and vanillin-based polymers was confirmed by FT-IR spectroscopy. As an example, the FT-IR spectra of pure DGEVADMA, HPPA, and polymer 20D/80H are shown in Figure S1. The intensity of the C=C group signal, which was present at 1636 cm^–1^ in the FT-IR spectra of DGEVADMA and HPPA, was reduced in the polymer spectrum. This indicated the formation of a polymer network. The intensities of the OH and C=O groups at 3454 and 1713 cm^–1^ remain constant in the polymer spectrum in comparison with the spectra of the starting materials, which is essential for the transesterification reaction.

### 3.3 Stress Relaxation of the Vitrimer

**Figure 3:**
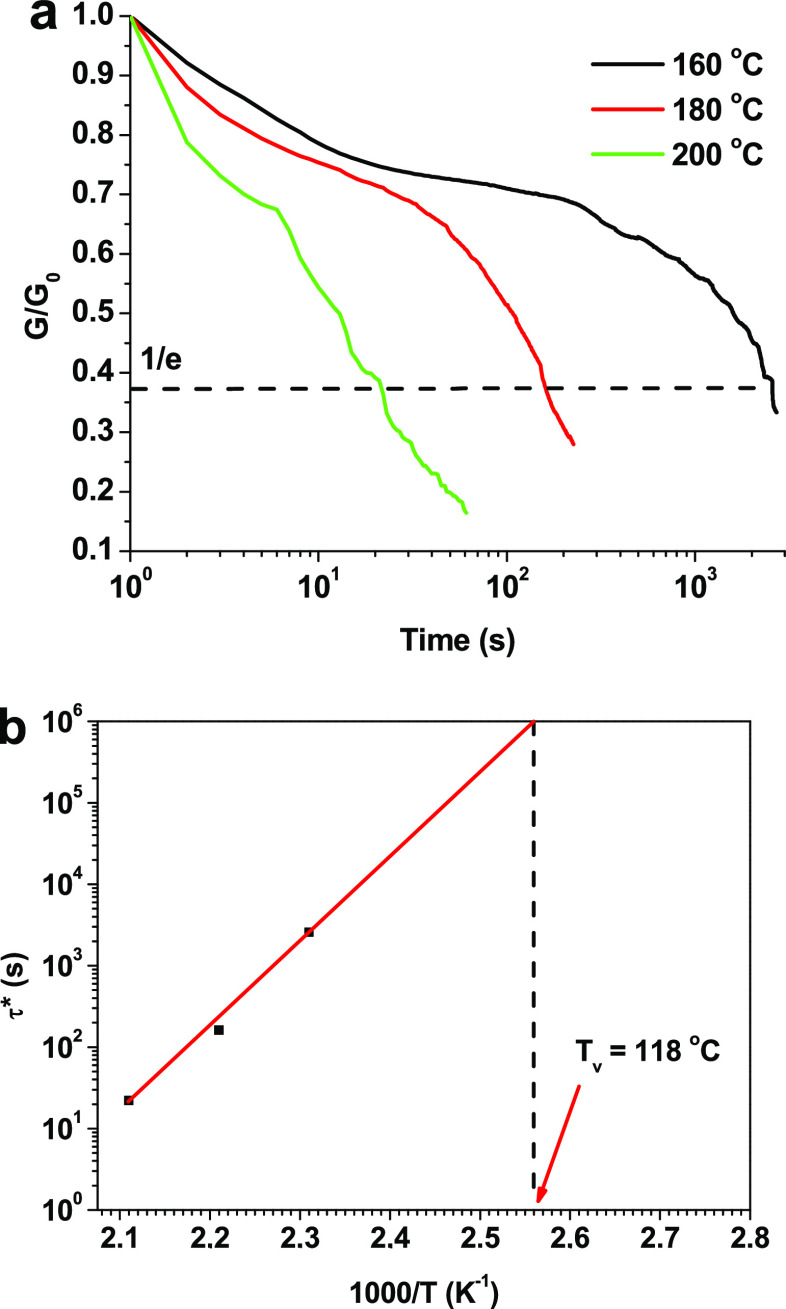
Stress relaxation curves versus time of the 20D/80H (a) and Arrhenius plot of relaxation times (b).

The dynamic bonds of the vitrimers enable the topology rearrangement of the network and alleviate the relaxation of internal stress caused by deformation.^34^ Meanwhile, thermosets have difficulties in stress relaxation as having permanent covalent bonds. To evaluate the rearrangement of polymer 20D/80H, stress relaxation tests were performed at temperatures of 160–200 °C (Figure 3). Relaxation time (τ*) can be obtained from stress relaxation curves and is defined as the time when the sample relaxes to 1/e of the initial modulus.^35^ As shown in Figure 3a, the photocured polymer 20D/80H can relax stress in the temperature range of 160–200 °C and τ* decreased from 43 min to 22 s due to dynamic bond exchange and chain diffusion. Vitrimers have two characteristic glass transition temperatures *T*_g_ and *T*_v_.^36^ Below *T*_v_, vitrimers behave as thermosets, while above *T*_v_, the exchange reactions speed up, making flow possible due to reversible reactions. *T*_v_ depends on the transesterification catalyst and its concentration and can be determined by extrapolating the data to a relaxation time of 10^6^.^36^ Therefore, the *T*_v_ of the vitrimer 20D/80H was determined from the Arrhenius curves and was 118 °C (Figure 3b).

### 3.4 Thermal Properties

The thermal properties of the polymers based on glycerol and vanillin were analyzed by DMTA and TGA, and the corresponding data are presented in Table 3. The *T*_g_ values (12–50 °C) were comparable to those of DGEVADMA polymers with dithiol (17–40 °C).^28^ Incorporation of HPPA into the network reduced the *T*_g_ since the neat polymer 100D had a *T*_g_ value of 50 °C, and the polymers with the HPPA fragment had values of 12–43 °C. This was due to the plasticizing effect of the glycerol fragment in the structure of HPPA caused by lower cross-linking density and branched structure. No significant correlation of the HPPA amount on the thermal properties was noticed. However, the polymer 20D/80H had the lowest values of *T*_g_, *T*_dec-10%_, and the char yield (12 °C, 283 °C, and 18%, respectively) due to the less cross-linked structure of the network, which was demonstrated by the yield of the insoluble fraction (95.0%). The other polymers had higher thermal stability due to the aromatic content, which is indicated by the char yield. The storage modulus versus temperature curves (Figure S2a) have a peak, indicating that the photocured samples might undergo crystallization, which is confirmed by the second peak/shoulder in the curves of tan δ (Figure S2b).^37,38^ The thermal stability of polymers is shown in thermogravimetric curves (Figure S2c), which have one step that confirms a densely cross-linked network.

**Table 3:** Yield of Insoluble Fraction and Thermal Characteristics of Polymers

		DMTA		TGA	
polymer	yield of insoluble fraction [%]^a^	*T*_g_ [°C]^b^	*G*_r_′ [MPa]^c^	*T*_dec-10%_ [°C]^d^	char yield [%]^e^
100D	97.0 ± 0.0	50	0.30	325	27
80D/20H	94.8 ± 0.9	30	0.09	327	25
60D/40H	97.7 ± 0.1	22	0.08	328	23
40D/60H	96.9 ± 0.4	43	0.24	328	21
20D/80H	95.0 ± 0.1	12	0.13	283	18

a After 24 h of Soxhlet extraction with acetone.

b Glass transition temperature determined by DMTA.

c Storage shear modulus of cured resins in the rubbery plateau region.

d Temperature at a weight loss of 10% obtained from TGA curves.

e From TGA curves.

### 3.5 Mechanical Properties

**Figure 4:**
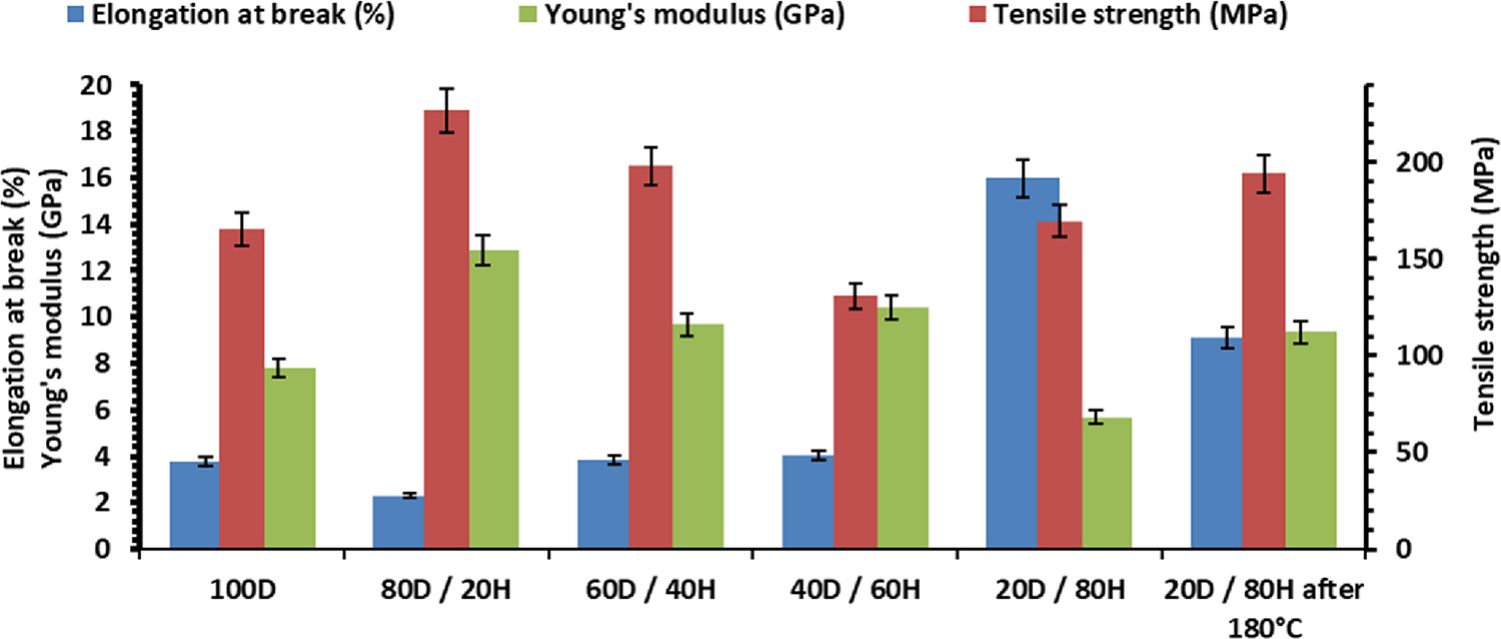
Mechanical characteristics of glycerol- and vanillin-based polymers.

The mechanical properties of glycerol- and vanillin-based polymers were investigated by tensile testing. Mechanical characteristics of photocured resins and polymer 20D/80H after welding and curing at 180 °C are presented in Figure 4. The incorporation of the monomer with the glycerol fragment provided a plasticizing effect due to lower cross-linking density and branched structure, which is seen by increased values of elongation at break. The polymer without HPPA had an elongation at a break of 3.78%, while polymers with HPPA had values of 2.31–15.97%, which were comparable to that of the DGEVADMA polymer with dithiol (12.3%).^28^ The amount of HPPA did not show a significant correlation with the mechanical characteristics. However, the polymer 20D/80H had higher values of Young’s modulus, tensile strength, and elongation at break than polymer 100D, which showed a denser cross-linked structure that correlates with storage modulus *G*′ values from photorheometry studies.

### 3.6 Self-Healing Properties

**Figure 5:**
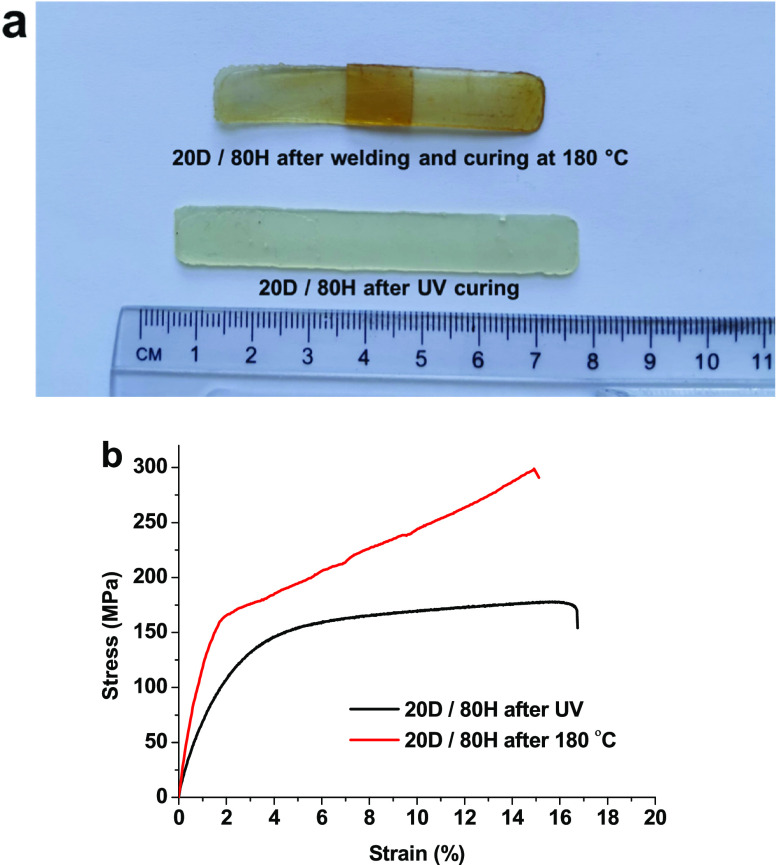
Photograph (a) and stress–strain curves (b) of sample 20D/80H after UV curing and repairing at 180 °C for 1 h.

The self-healing properties were investigated by cutting the photocured sample 20D/80H, rejoining at 180 °C for 1 h, and mechanically testing by a tensile experiment (Figure 5). The healed sample exhibited higher Young’s modulus and tensile strength values than the original sample (Figure 5b). Young’s modulus of the thermally treated sample was 1.5 times higher than the photocured sample, and the welding efficiency of tensile strength was up to 114.12%. The reason may be the occurrence of dynamic transesterification reactions that result in the repair of the damaged sample. In addition, the photocured sample could not be fully cured, and at higher temperatures, residual carboxyl, C=C, and hydroxyl groups can lead to further reactions.

### 3.7 DLP 3D Printing and Shape-Memory Properties

**Figure 6:**
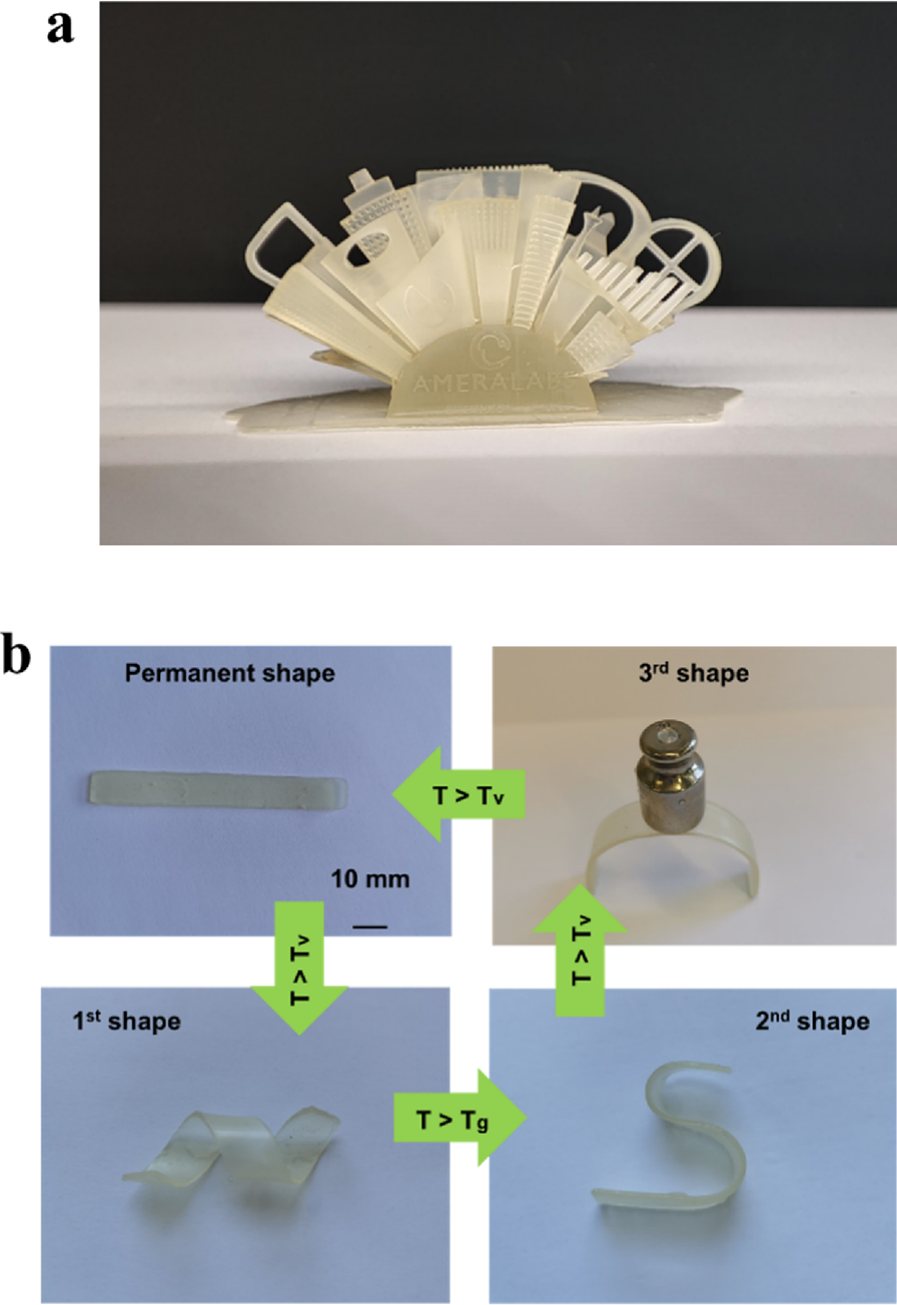
Photographs expressing DLP printing of a complex “City” structure (a) and monitoring the shape-memory behavior of the polymer 20D/80H sample (b).

To demonstrate the suitability of the resin 20D/80H for DLP 3D printing, a complex “City” structure was formed (Figure 6a). The DLP printed object showed high printing accuracy of small details with smooth surface finishing.

Shape-memory polymers can be applied as artificial muscles and actuators due to reversible actuation as they can transit between shapes spontaneously and reversibly when heating or cooling is applied.^39^ Therefore, the monitoring of the shape-memory behavior of the polymer 20D/80H is presented in Figure 6b. The sample was heated above *T*_g_ or *T*_v_, transformed to the desired temporary shape by applying external force, cooled down, and fixed for a short period of time. The first shape was fixed by cooling the sample above *T*_g_ to 40 °C. The second and third shapes were obtained by cooling the sample below room temperature. The third shape was capable of holding a weight of 20 g. After being heated above the *T*_v_, the sample completely recovered to the permanent shape, showing an excellent shape-memory property, due to the free hydroxyl groups of monomers that provided plasticity.

### 3.8 Recyclability

**Figure 7:**
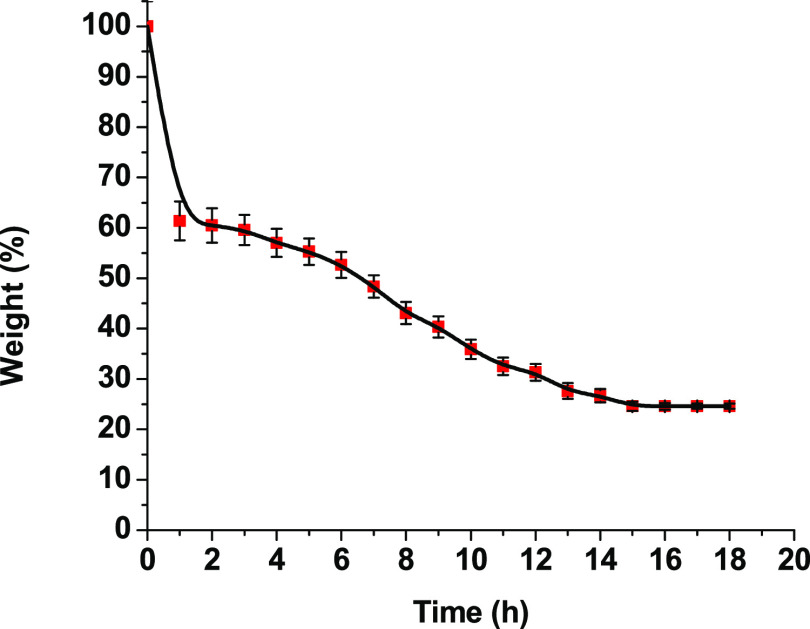
Weight loss of the polymer 20D/80H sample versus temperature.

The recycling rate of plastic waste is less than 10% due to resistance to degradation and distribution in industry.^40^ The degradation of polymeric materials would reduce the negative impact on the environment. Vitrimers can be chemically recycled by alcoholysis at elevated temperatures as a result of dynamic transesterification reactions between the ester and hydroxyl groups. Therefore, the alcoholysis of the sample was carried out with ethanol and the weight loss of 20D/80H is presented in Figure 7. The weight of the sample decreased, showing that the transesterification reaction between the ester bonds of the sample and hydroxyl groups of ethanol occurred as the cross-linked network was deconstructed, and the degraded product was dissolved in ethanol. The sample lost weight rapidly to 61% after 1 h and gradually decreased to 25% during 15 h and stabilized. The residue was left as a result of permanent cross-links of the sample. Notably, the intensities of the OH, C=C, and C=O group signals at 3457, 1636, and 1713 cm^–1^ remained the same in the spectra of the sample 20D/80H residue after alcoholysis and dissolved in ethanol since the rearrangement of the cross-linked network occurred via dynamic transesterification (Figure S3).

## 4 Conclusions

Glycerol- and vanillin-based vitrimers have been designed and synthesized using an environmentally friendly strategy by combining renewable resources, UV-curing, and dynamic transesterification reactions. DGEVA dimethacrylate was used for the synthesis of vitrimers by photocuring for the first time. The resin with the highest amount of functionalized glycerol was selected for vitrimer synthesis due to the viscosity suitable for optical 3D printing and the highest amount of hydroxyl and ester groups that are beneficial for transesterification reactions. The dynamic vitrimer with a weight ratio of 8:2 of glycerol- and vanillin-based monomers had a topology freezing temperature of 118 °C. The self-healing properties were exhibited as a result of plasticity provided by flexible chains and free hydroxyl groups. The appearance of dynamic transesterification and reactions of the residual groups imparted the self-healing and welding efficiency of the tensile strength up to 114.12%. The cross-linked network was deconstructed as a result of the transesterification reaction between the ester bonds of the sample and hydroxyl groups of ethanol, and a 75% efficiency was reached. The synthesized vitrimer could contribute to environmental protection as it is partially recyclable, can be healed due to weldability, can change the permanent shape to the desired one, and can be used in reversible actuation as artificial muscles and actuators, where transits between two shapes are required. The photocurable resin designed on the basis of glycerol and vanillin is a promising resin for the optical 3D printing of vitrimers on demand.

## Supporting Information Available

FT-IR spectra of DGEVADMA, HPPA, and cross-linked polymer 20D/80H; curves of the storage modulus *G*′ versus temperature; curves of tan δ versus temperature; thermogravimetric curves of glycerol- and vanillin-based polymers; and FT-IR spectra of the sample 20D/80H residue after alcoholysis and dissolved in ethanol (PDF)
