# Supplementary material for: Photocurable Glycerol- and Vanillin-Based Resins for the Synthesis of Vitrimers
Source: ACS Appl Polym Mater. 2022 Aug 2;4(8):6103–10. doi: 10.1021/acsapm.2c00914 (PMC9379905; doi:10.1021/acsapm.2c00914)
Supplement: Supplementary file 1 — ap2c00914_si_001.pdf [file ap2c00914_si_001.pdf]

Supporting information for

Photocurable Glycerol and Vanillin-based Resins for the Synthesis of Vitrimers

*Sigita Grauzeliene[1], Marius Kastanauskas[1], Vaidas Talacka[2], Jolita Ostrauskaite[1]**

[1] Department of Polymer Chemistry and Technology, Kaunas University of Technology, Radvilenu Rd. 19, Kaunas LT-50254, Lithuania

[2] AmeraLabs, Kestucio str. 6A, Kaunas LT-44320, Lithuania

Corresponding Author
Jolita Ostrauskaite − Email: jolita.ostrauskaite@ktu.lt

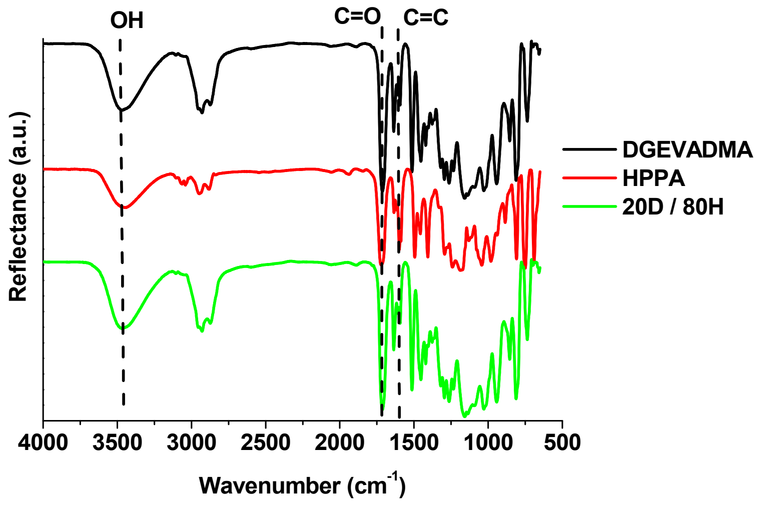

Figure S1. FTIR spectra of DGEVADMA, HPPA, and cross-linked polymer 20D / 80H.

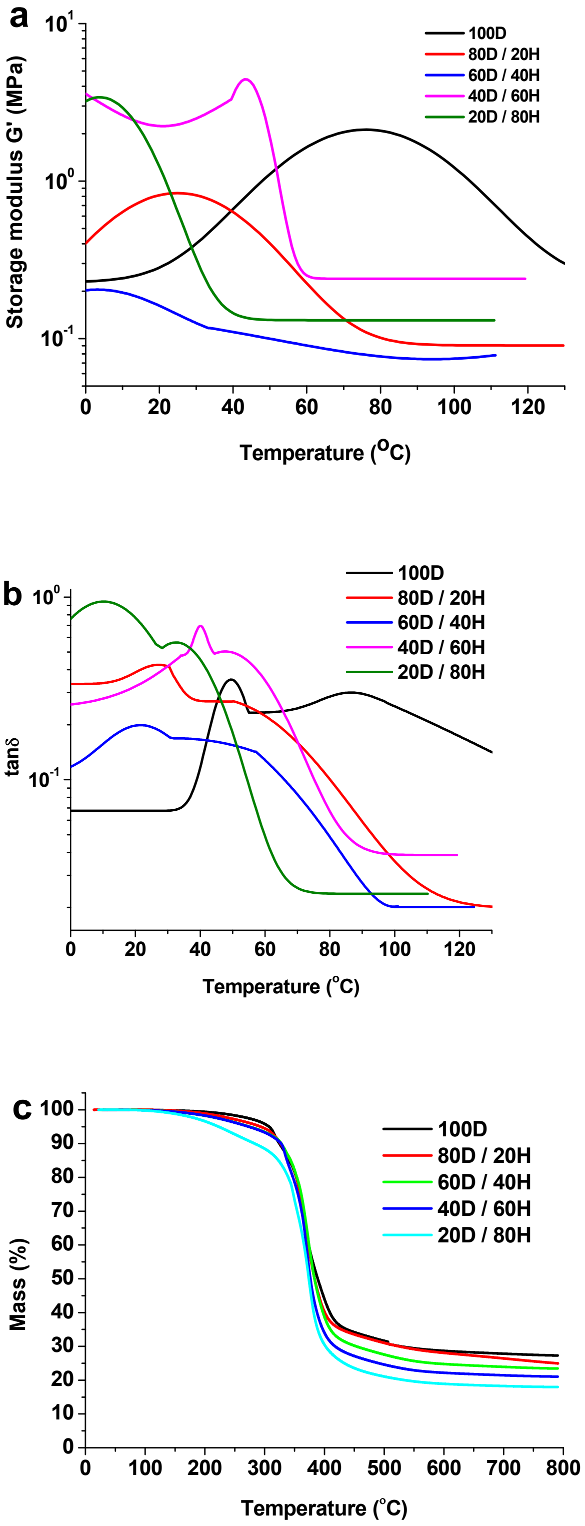

Figure S2. Curves of the storage modulus G' versus temperature (a), curves of tanδ versus temperature (b), and thermogravimetric curves of glycerol and vanillin-based polymers (c).

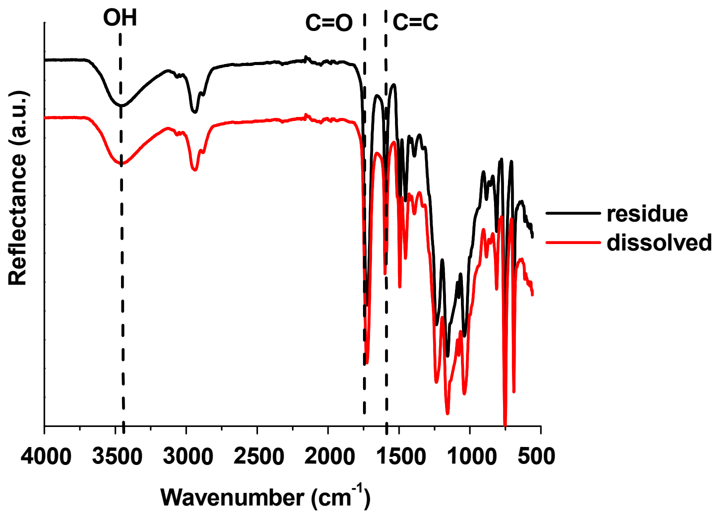

Figure S3. FTIR spectra of the sample 20D / 80H residue after alcoholysis and dissolved in ethanol.
